# Vegetation Responses to Climate Change and Anthropogenic Activity in China, 1982 to 2018

**DOI:** 10.3390/ijerph19127391

**Published:** 2022-06-16

**Authors:** Jie Li, Mengfei Xi, Lijun Wang, Ning Li, Huawei Wang, Fen Qin

**Affiliations:** 1 College of Geography and Environmental Science, Henan University, Kaifeng 475004, China; leejie@henu.edu.cn (J.L.); xmf@henu.edu.cn (M.X.); henuwlj@henu.edu.cn (L.W.); lining@henu.edu.cn (N.L.); wanghuawei_gengen@163.com (H.W.); 2 Key Laboratory of Geospatial Technology for the Middle and Lower Yellow River Regions, Ministry of Education, Henan University, Kaifeng 475004, China; 3 Henan Industrial Technology Academy of Spatio-Temporal Big Data, Henan Univesity, Kaifeng 475004, China; 4 Henan Technology Innovation Center of Spatial-Temporal Big Data, Henan University, Kaifeng 475004, China

**Keywords:** GIMMS NDVI3g, MOD13A2, climate change, human activity, normalized difference vegetation index

## Abstract

Climate change and human activities significantly affect vegetation growth in terrestrial ecosystems. Here, data reconstruction was performed to obtain a time series of the normalized difference vegetation index (NDVI) for China (1982–2018) based on Savitzky–Golay filtered GIMMS NDVI3g and MOD13A2 datasets. Combining surface temperature and precipitation observations from more than 2000 meteorological stations in China, Theil–Sen trend analysis, Mann–Kendall significance tests, Pearson correlation analysis, and residual trend analysis were used to quantitatively analyze the long-term trends of vegetation changes and their sources of uncertainty. Significant spatial and temporal heterogeneity was observed in vegetation changes in the study area. From 1982 to 2018, the vegetation showed a gradually increasing trend, at a rate of 0.5%·10 a^−1^, significantly improving (37.15%, *p* < 0.05) more than the significant degradation (7.46%, *p* < 0.05). Broadleaf (0.66) and coniferous forests (0.62) had higher NDVI, and farmland had the fastest rate of increase (1.02%/10 a^−1^). Temperature significantly affected the vegetation growth in spring (R > 0; *p* < 0.05); however, the increase in summer temperatures significantly inhibited (R < 0; *p* < 0.05) the growth in North China (R_NDVI-tem_ = −0.379) and the Qinghai–Tibetan Plateau (R_NDVI-tem_ = −0.051). Climate change has highly promoted the growth of vegetation in the plain region of the Changjiang (Yangtze) River (3.24%), Northwest China (1.07%). Affected by human activities only, 49.89% of the vegetation showed an increasing trend, of which 22.91% increased significantly (*p* < 0.05) and 9.97% decreased significantly (*p* < 0.05). Emergency mitigation actions are required in Northeast China, Xinjiang, Northwest China, and the Qinghai–Tibetan Plateau. Therefore, monitoring vegetation changes is important for ecological environment construction and promoting regional ecological protection.

## 1. Introduction

The recent sixth assessment report of the United Nations Intergovernmental Panel on Climate Change (IPCC) revealed that climate change and anthropogenic activities have significantly shaped the fundamental system processes of the planet [1]. Accordingly, monitoring vegetation change and attribution analyses have become an important part of environmental research [2,3]. Vegetation plays an essential role in the global hydrological and carbon cycles, as well as overall energy exchange [4,5,6]; therefore, the impact of global climate change on vegetation growth has received increasing attention over recent decades.

Under modern climate change, quantitative studies of the relationship with vegetation become critical at regional and global scales [7,8]. Vegetation growth is affected by various factors, among which precipitation and temperature are generally considered the most influential climatic variables [9]. Conventionally, vegetation thrives in areas with ample water and heat, growing sparsely in drier areas with limited rainfall [10,11,12,13]. Chu et al. analyzed the response of vegetation to climate in the Amur–Heilongjiang River Basin from 1982 to 2015, and found that NDVI in the growing season was mainly affected by precipitation, and temperature was the dominant factor affecting vegetation growth in spring [14]. Gao et al. found that precipitation was the most important factor affecting the variation of NDVI in the Three-River Headwater Region [15]. Yao et al. found that annual precipitation was the first dominant factor affecting vegetation growth in Inner Mongolia [16]. Chen et al. studied the impact of climate change and human activities on grasslands in Central Asia, and found that precipitation was the main climatic factor affecting grassland dynamics in Central Asia, and overgrazing accelerated grassland degradation [17]. Moreover, anthropogenic activities exert both promoting and inhibiting effects on vegetation cover change. For instance, rapid urbanization has led to the encroachment of construction into tremendous amounts of farmland and forest, thereby significantly reducing the vegetation cover [14]. In contrast, the implementation of vegetation construction projects, such as afforestation or restoring farmland to forests and grasslands, increases coverage [18,19]. Therefore, both human activity and climate change influence vegetation cover, yielding significant regional differences around the globe. Therefore, large-scale and long-term vegetation change monitoring and attribution analyses can serve as a reference for formulating reasonable land use and ecological protection protocols.

The normalized difference vegetation index (NDVI) is an effective method for reflecting vegetation growth in the field of remote sensing [4]. The index is widely used for detecting the interactions between vegetation and climate change [19,20], land use [21], crop classification [22], and forest monitoring [23]. SPOT-VGT, AVHRR (Advanced Very High-Resolution Radiometer), and MODIS (Moderate Resolution Imaging Spectroradiometer) from different sensors form where NDVI can be derived, although their precise spectral response functions, calibration and data processing methods, synthesis time, and spatiotemporal resolutions vary [24,25]. It is difficult to effectively capture long time series of surface processes by relying solely upon certain satellite products, although multisource data fusion algorithms can make this possible [21]. The AVHRR GIMMS NDVI3g dataset is reportedly the best data source to represent vegetation, as it comprises a long time series with global coverage and a strong ability to represent vegetation change dynamic changes [26]. However, the time resolution of GIMMS is 1982 to 2015, and the research duration is greatly limited, making it difficult to carry out new research [27]. Thus, supplementing it with other remote sensing data sources can greatly extend its lifetime. As the spatiotemporal resolutions of MOD13A2 are superior to that of GIMMS, MOD13A2 can be readily combined with GIMMS image data [28,29]. Due to the large-signal noise present in the GIMMS NDVI3g data, Savitzky–Golay (S-G) filtering is used to reconstruct the time series by effectively removing outliers, better preserving the continuity of vegetational changes [30]. Therefore, merged GIMMS and MODIS data can generate NDVI datasets more capable of exploring long-term vegetation evolution characteristics [31,32].

Due to the limited time range of GIMMS data, the spatiotemporal patterns of long-term vegetation responses and feedback to climatic factors across different regions of China remain unclear. Accordingly, this study extended the GIMMS-derived NDVI time series by merging it with MODIS datasets. Moreover, the dynamic evolution of vegetation and its coupled relationship with influencing factors were quantitatively analyzed across different spatiotemporal scales throughout the country. It is intended that the findings presented here will serve as a reference for China’s environmental protection and sustainable development.

## 2. Materials and Methods

### 2.1. Study Area

**Figure 1:**
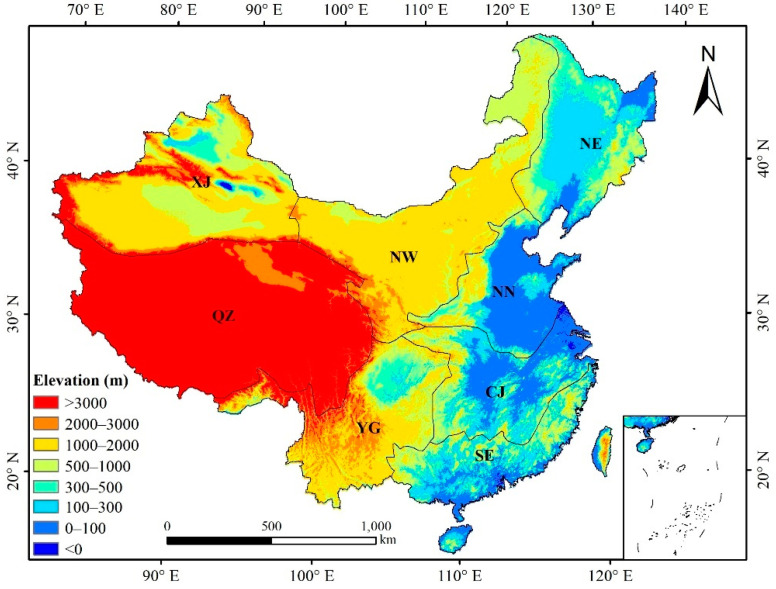
Eight subregions of the study area and their elevation: Xinjiang (XJ), Qinghai–Tibetan Plateau (QZ), Northwest China (NW), Northeast China (NE), North China (NN), southwest Yungui Plateau (YG), the plains region of Changjiang (Yangtze) River (CJ), and Southeast China (SE).

China spans a vast area of ~9.6 million km^2^, spread over 34 provincial administrative regions. China has the most extensive monsoon climate region, ranging from a temperate continental climate to the extreme north to a temperate monsoon climate in the northeast, a subtropical monsoon climate in the extreme south, and a highland mountain climate in the Tibetan Plateau [32]. The annual precipitation in China increases gradually from the northwest to the southeast. The southeast of China is primarily semihumid and humid, whereas the northwest is mainly arid and semiarid [20]. This study divides China into eight subregions based on elevation, precipitation, and so on (Figure 1).

### 2.2. Data Source

#### 2.2.1. NDVI Dataset

(1) GIMMS NDVI3g

This study used the most recent (third) generation NOAA/AVHRR GIMMS data from the NASA Global Monitoring and Modeling Research Group (https://nex.nasa.gov/nex, accessed on 10 June 2021). The spatiotemporal resolution of the GIMMS NDVI3g dataset is 15 d and 8 km (0.08333°). The GIMMS NDVI3g dataset covers the world in space, with a time span from 1982 to 2015, and is currently the NDVI data product with the longest time range. Specifically, the GIMMS NDVI3g dataset was designed to improve data quality [33].

(2) MODIS NDVI

The MODIS NDVI dataset, derived from the Terra satellite (https://earthdata.nasa.gov/, accessed on 10 June 2021), was used here to bolster the integrity of the time series from 2001 to 2018. It maintains a temporal resolution of 16 d and a spatial resolution of 1 × 1 km. Image extraction, projection conversion, mosaicking, cropping, and other preprocessing steps were performed using the image-based on Python script. To ensure consistent spatial resolution, the MOD13A2 dataset was resampled to 8 × 8 km. The maximum value composite method (MVC) was used to synthesize the remote sensing data into monthly values, and mean values were used to synthesize monthly vegetation coverage into annual estimates. The GIMMS and MODIS NDVI after Savitzky–Golay filtering are respectively defined as GIMMS_S-G_ and MODIS_S-G_.

#### 2.2.2. Meteorology

The China Meteorological Data Sharing Network (http://data.cma.cn/, accessed on 1 July 2021) provides daily temperature and precipitation data from more than 2000 meteorological stations. These data were used to construct a raster dataset of monthly values across China from 1982 to 2018 via local thin-plate spline interpolation. Daily temperature and precipitation data were averaged and synthesized into annual data before resampling to 8 × 8 km for further analysis with the NDVI time series.

Due to the use of meteorological data of more than 2000 stations provided by the China Meteorological Administration, the current data can only be collected and obtained in the latest year from 1961 to 2018. Limited by data availability, the data source time from 1982 to 2018 should be selected given that GIMMS NDVI data can only be obtained from 1982 at the earliest.

### 2.3. Data Postprocessing and Methodology

#### 2.3.1. NDVI Data Postprocessing

Due to inherent differences in the spatiotemporal resolutions of satellite sensors, it was necessary to examine correlations before and after Savitzky–Golay filtering prior to merging the two datasets. Based on the MATLAB platform, this study carries out pixel-by-pixel correlation analysis and regression analysis on GIMMS and MODIS. The correlation coefficient can be calculated according to Equation (1):

(1) $$R=\frac{cov\left(G,M\right)}{\sqrt{var\left(G\right)var\left(M\right)}},$$

where *R* is the correlation coefficient, *G* and *M* are GIMMS and MOD13A2 monthly NDVI from 2001–2015, and *var* is the variance function. The covariance between *G* and *M* is denoted by *cov*(*G*, *M*).

Next, pixelwise linear regression was performed on GIMMS and MOD13A2 NDVI monthly data, and the 2016–2018 NDVI data were extended using the pixel-by-pixel linear regression equation (Equations (2)–(4)):

(2) $$k=\frac{n\sum_{i=1}^{n}MODIS_{i}GIMMS_{i}-\sum_{i=1}^{n}MODIS_{i}\sum_{i=1}^{n}GIMMS_{i}}{n\sum_{i=1}^{n}MODIS_{i}^{2}-{\left(\sum_{i=1}^{n}MODIS_{i}\right)}^{2}},$$

(3) $$\;b=\bar{GIMMS}-k\times \bar{MODIS}$$

(4) $$GIMMS_{i}=k\times MODIS_{i}+b+\varepsilon_{i},$$

where $GIMMS_{i}$ and $MODIS_{i}$ represent the corresponding GIMMS and MOD13A2 NDVI values in the *i*th month, respectively; *n* is the total number of months; $\varepsilon_{i}$ represents random error; and $\bar{GIMMS}$ and $\bar{MODIS}$ are the mean values of the monthly NDVI for the corresponding pixels from 2001 to 2015. The 2016–2018 GIMMS NDVI fitted by MODIS is represented by GIMMSMOD data (2016–2018). In this way, a new NDVI was constructed to provide data support for further research.

#### 2.3.2. Trend Analysis and Significance Tests

The Theil–Sen median method (capable of reflecting trend changes) and the Mann–Kendall (M–K) method (which assesses the significance of trend changes) were used for vegetation trend analysis over long time series [16,34]. Compared with the common linear regression method for assessing interannual variations in vegetation changes, the methods proposed here have a stronger ability to avoid data error and reduce the influence of outliers while increasing the accuracy and reliability of the analytical test results. The Theil–Sen median method was calculated according to Equation (5):

(5) $$\beta =median\left(\frac{NDVI_{i}-NDVI_{j}}{i-j}\right),\forall \;i<j,$$

where *β* is the NDVI time series. When *β* > 0, it reflects the increasing trend, whereas *β* < 0 indicates a decreasing trend.

The M–K significance tests used to identify significant time series trends have been successfully employed in other similar studies of hydrology and meteorology, proving themselves advantageous in that samples are not required to follow a normal distribution and are not overly influenced by outliers. The appropriate formula for calculating M–K is presented in Equations (6)–(9):

(6) $$Z_{c}=\{\;\begin{array}{cc} \frac{S-1}{\sqrt{var\left(S\right)}} & S>0\\ 0 & S=0\\ \frac{S+1}{\sqrt{var}} & S<0 \end{array}$$

where

(7) $$S=\sum_{i=1}^{n-1}\sum_{k=i+1}^{n}sign\left(NDVI_{k}-NDVI_{i}\right),$$

(8) $$var\left(S\right)=\frac{n\left(n-1\right)\left(2n+5\right)-\sum_{i=1}^{n}t_{i}\left(t_{i}-1\right)\left(2t_{i}+5\right)}{18},$$

(9) $$sign\left(NDVI_{k}-NDVI_{i}\right)=\{\;\begin{array}{cc} 1 & NDVI_{k}-NDVI_{i}>0\\ 0 & NDVI_{k}-NDVI_{i}=0\\ -1 & NDVI_{k}-NDVI_{i}<0 \end{array}$$

where *NDVI_k_* and *NDVI_i_* are the NDVI datasets in the *k*th and *i*th years, respectively, and the length of the study object is *n*. Here, the M–K significance tests were performed as follows: The null hypothesis H0 was *β* = 0 at a given significance level of α = 0.05, and |Zc| > 1.96 was the threshold for satisfying the significance test.

#### 2.3.3. Pearson Correlation Analysis

The Pearson correlation analysis method was performed to calculate the correlation coefficient (CC) between NDVI, temperature, and precipitation over the analysis period (1982–2018), in accordance with Equation (10):

(10) $$R_{ab}=\frac{\sum_{i=1}^{n}\left(a_{i}-\bar{a}\right)\left(b_{i}-\bar{b}\right)}{\sqrt{\sum_{i=1}^{n}{\left(a_{i}-\bar{a}\right)}^{2}}\sqrt{\sum_{i=1}^{n}{\left(b_{i}-\bar{b}\right)}^{2}}},$$

where *R_ab_* is the CC; *n* is the number of samples; *a_i_* and bi denote the values of variables *a* and *b* in the year *i*, respectively; $\bar{a}$ and $\bar{b}$ represent the average of variables *a* and *b*, respectively.

#### 2.3.4. Residual Trend Analysis

Although long-term observational data regarding the effects of anthropogenic activities on vegetation (i.e., NDVI) are severely lacking, the effects related to human factors and climate change can be distinguished via residual analysis methods [35]. Here, the independent variables are temperature and precipitation, with vegetation status as the dependent variable, and multiple linear regressions (MLRs) were used to fit the predicted NDVI (NDVI_pre_) [36]. Residuals were obtained by calculating the differences between satellite sensor-derived NDVI and NDVI_pre_ [36], where positive (negative) trends indicated that human activities play a positive (deleterious) role [27].

## 3. Results

### 3.1. Precision Validation

**Figure 2:**
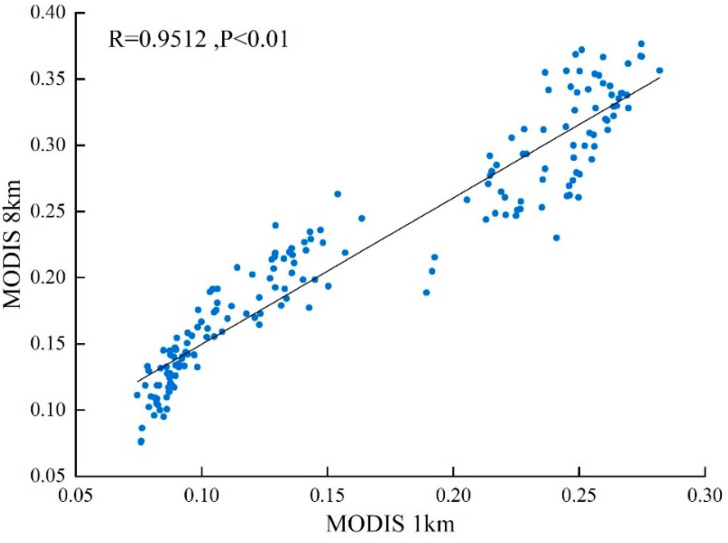
Correlation analysis and linear fitting of MODIS data before and after resample (*p* < 0.01).

The GIMMS and MOD13A2 remote sensing data sensors notably differ in terms of band number, wavelength, and spatiotemporal resolution, each of which requires assessment for consistency (Table 1). Correlation analysis and linear fitting of MODIS data were performed before and after resample, and found that the data were highly consistent (R = 0.9512, *p* < 0.01) (Figure 2). Here, ${\mathrm{GIMMS}}_{\mathrm{MOD}}$ data (2016–2018) and ${\mathrm{GIMMS}}_{S-G}$ (1982–2015) were selected to construct a raster dataset of vegetation cover in China from 1982 to 2018. From 2001 to 2015, the CC between GIMMS and MOD13A2 was 0.941, followed by GIMMS_S-G_ and MOD13A2 (0.929). The CC between GIMMS and MODIS_S-G_ was notably higher (0.962); however, that between GIMMS_S-G_ and MOD13A2_S-G_ reached a maximum of 0.966 (*p* < 0.01, *n* = 180), indicating that S–G filtering helped to remove or reduce noise and outliers contained in data, thereby effectively improving image quality. When calculating the correlation between the numerical simulation ${\mathrm{GIMMS}}_{\mathrm{MOD}}$ (2001–2015) and the same period ${\mathrm{GIMMS}}_{S-G}$, the results showed that monthly and pixelwise correlations from 2001 to 2015 between ${\mathrm{GIMMS}}_{S-G}$ and ${\mathrm{GIMMS}}_{\mathrm{MOD}}$ were 0.98 (*p* < 0.01) and 0.91, respectively, among which 91% of the pixels were significant at the α = 0.05 level, thus confirming the quality of remote sensing fusion data. Product accuracy reliability was thus deemed suitable for the dynamic study of vegetation cover in China. The verification results showed that the reconstruction models employed here could effectively reduce the deviation between AVHRR and MODIS NDVI data; therefore, the reconstructed MODIS data can supplement the temporal limitations of the AVHRR NDVI data time series.

### 3.2. Spatiotemporal Variations

#### 3.2.1. Temporal Trends of Vegetation, 1982–2018

**Figure 3:**
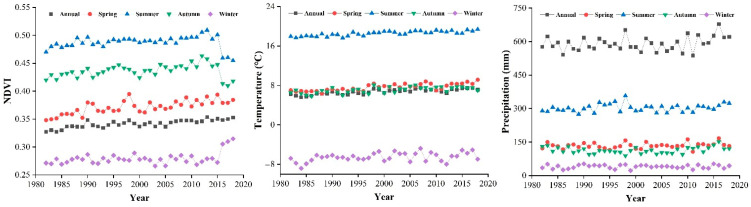
Variation of NDVI, temperature, and precipitation over the analysis period, 1982–2018.

Figure 3 shows the annual variation of NDVI over the study area from 1982 to 2018, as well as by season. The largest average NDVI value was in 2013 (0.354), whereas the minimum was in 1984 (0.327), with an overall average of 0.342. NDVI values were significantly higher in summer (0.49), followed by autumn (0.44), spring (0.37), and winter (0.28). The NDVI showed an increasing trend of the analysis period, with growth rates ranking in the following order: spring (0.8% × 10 a^−1^) > annual (0.5% × 10 a^−1^) > winter (0.4% × 10 a^−1^) > autumn (0.3% × 10 a^−1^) > summer (0.06% × 10 a^−1^).

**Figure 4:**
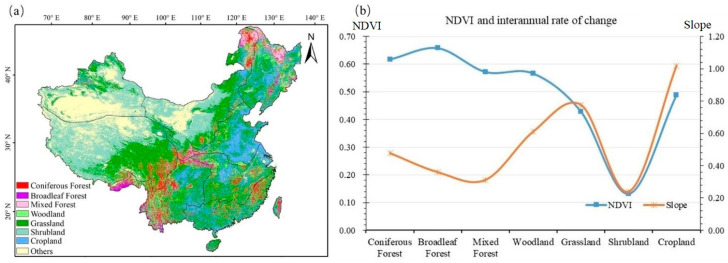
(**a**) Spatial distribution of vegetation types. (**b**) NDVI and interannual rate of change for vegetation types.

With reference to the main vegetation composition in China [14], Figure 4a shows the map of vegetation types in the study area, whereas Figure 4b shows the multiyear mean NDVI values and the corresponding slopes of change for each vegetation type. From the NDVI values of different vegetation types (Figure 4b), broadleaf and coniferous forests had the highest NDVI (0.66 and 0.62, respectively), followed by mixed forests and woodlands (both 0.57), farmlands (0.49), grasslands (0.43), and shrublands (0.13). In terms of the slope of change in NDVI by vegetation type (Figure 4b), all showed an increasing trend, with farmland NDVI increasing the fastest (1.02%·10 a^−1^), followed by grassland (0.78%·10 a^−1^), coniferous forests (0.48%·10 a^−1^), broadleaved forests (0.36%·10 a^−1^), and shrublands (0.24%·10 a^−1^).

#### 3.2.2. Vegetation Spatial Change Trend

**Figure 5:**
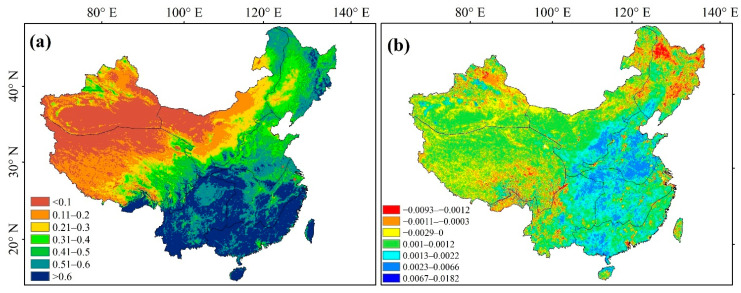
Vegetation interannual variation trend: (**a**) mean NDVI; (**b**) NDVI variation trend.

The spatial distribution of average NDVI showed significant spatial heterogeneity across China from 1982 to 2018 (Figure 5a). Overall, NDVI gradually increased from the northwest to the southeast, with peak values mostly distributed in NN, YG, CJ, SE, southeast QZ, Daxing’anling, and NE. Conversely, lower NDVIs were mainly concentrated in XJ, NW, and northwest QZ. The results of the Theil–Sen median trend analysis showed that vegetation gradually increased at a rate of 0.5%·10 a^−1^ across China, with trends increasing most rapidly (slope ≥ 1.3 × 10^−3^ a^−1^) mostly distributed in NN, CJ, NW Loess Plateau, and central YG and SE (Figure 5b). Alternatively, decreasing trends were strongest in XJ, the surrounding areas of NE, parts of QZ, and economically developed large urban agglomerations, largely due to slow decreases, as the slope trend rates mostly ranged from −9.3 × 10^−3^ to approximately 0 a^−1^.

Combining ground observations of the study area with research on arid and semiarid areas, the NDVI values across China were divided into seven grades (Table 2). NDVI > 0.1 accounted for 78.58% of the total study area; however, the overall vegetation coverage of the study area was low, with an average NDVI of only 0.34. NDVI > 0.4 accounted for 42.79% of the total area, with the vegetation type under this grade being dominated by forested land. A total of 16.5% of the total area was occupied by areas with NDVI > 0.6, and the vegetation types under this class were mainly grassland and cropland. The nonvegetated area (NDVI < 0.1) accounted for 21.72% of the total area, mainly composed of construction land and lakes, as well as the Gobi and other deserts.

#### 3.2.3. Spatial Patterns of Vegetation

**Figure 6:**
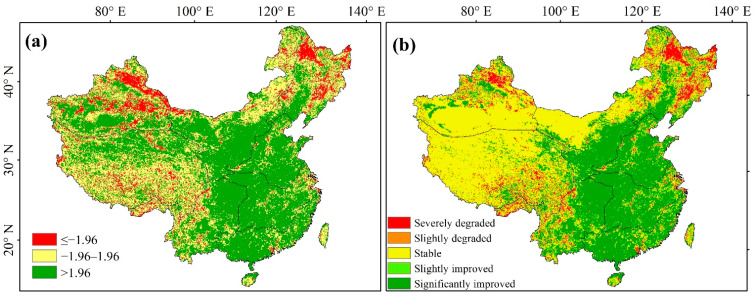
Spatial distribution of (**a**) M–K trend tests and (**b**) variation trend of the interannual NDVI in China from 1982–2018.

Combining the classification results from the Theil–Sen median analysis (Figure 6b) and the M–K trend test (Figure 6a), pixel-level NDVI trend data were obtained (Figure 6b), and the results were classified into five types: severely degraded, slightly degraded, stable, slightly improved, and significantly improved (Table 3). Table 3 and Figure 6b collectively indicate that the area of increasing vegetation in China from 1982 to 2018 was larger than that of degradation. Indeed, 40.15% showed an improved trend, with significantly improved NDVI (*p* < 0.05) accounting for 37.15% of this region, primarily distributed in NN, YG, CJ, SE, and south NW, notable areas with abundant water resources. Conversely, areas with degraded vegetation accounted for 9.34% of the study area, with severely degraded NDVI (*p* < 0.05) accounting for 7.46% of this region, and mostly distributed in NE, the Greater Khingan Range of NW, north XJ, and parts of southeast QZ, notably sparsely populated and water-scarce regions. The stable area (i.e., no significant change) is 50.52%, mostly located in south XJ, west QZ, and the Inner Mongolian Plateau in NW, largely representing the Gobi or other desert areas, as well as bare land.

**Figure 7:**
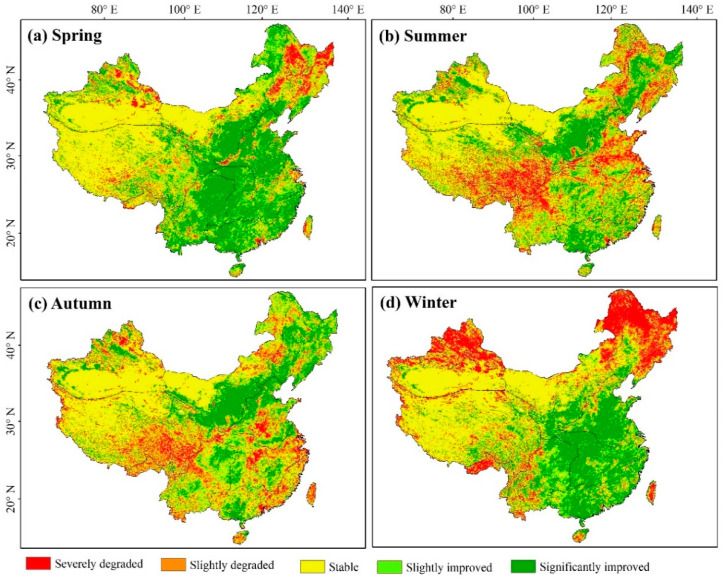
Seasonal variation trend in vegetation characteristics from 1982 to 2018.

Figure 7 shows the seasonal changes in vegetation from 1982 to 2018, where, again, the improved area of vegetation coverage was greater than the degraded area, and the spring vegetation growth was significantly larger than that of other seasons. The overall trend in spring was similar to that of the annual variation (Figure 6b). Indeed, the area with significantly improved NDVI values in spring accounted for 34.59% of the study region, primarily distributed in the central part of China. Notably, 5.98% of the total area was significantly degraded, mainly in NE and north XJ. The overall distribution of trends in summer and autumn were relatively similar, with significantly improved areas accounting for 21.85% and 22.73% and severely degraded areas accounting for 11.64% and 8.94%, respectively. Southeastern QZ showed a degradation trend in summer and autumn, whereas the southern part of NW showed a significant improvement. The trend of vegetation degradation is obvious in winter, with the significantly degraded region accounting for 17.42%, mostly distributed in NE, north XJ, and the Xiaoxing’an Mountains.

### 3.3. Analyses of Spatiotemporal Changes in Climate, 1982–2018

#### 3.3.1. Temporal Variation Characteristics

Figure 3 shows the temporal changes in temperature and precipitation from 1982 to 2018. The average annual temperature across China was 6.69 °C, peaking in 2007 (7.51 °C) and reaching its minimum in 1984 (5.57 °C). Average summer temperatures (18.55 °C) were significantly higher than those of other seasons, whereas winter temperatures represented the minimum (−6.53 °C). There were slight differences in spring, autumn, and average annual temperatures (7.62, 7.10, and 6.69 °C, respectively). From 1982 to 2018, temperatures showed an upward trend, where, yearly, spring, summer, autumn, and winter temperatures warmed at rates of 0.0395, 0.0535, 0.0369, 0.0339, and 0.0336° C·a^−1^, respectively.

From 1982 to 2018, the annual average precipitation was significantly higher than that of the four seasons; summer precipitation increased significantly, with the least precipitation being recorded in winter. The average annual precipitation in China was 591.02 mm, with the maximum observed in 2016 (678.54 mm) and the minimum recorded in 2011 (537.49 mm). Average seasonal precipitation varied greatly between spring (134.50 mm), summer (303.27 mm), autumn (113.71 mm), and winter (40.00 mm). Over the study period, an average increasing trend of annual precipitation of 0.805 mm·a^−1^ was observed, with those of spring, summer, autumn, and winter increasing at rates of 0.226, 0.340, 0.206, and 0.032 mm·a^−1^, respectively. Overall, with their more concentrated precipitation levels, spring and summer more remarkably contributed to the significant increase in annual precipitation.

#### 3.3.2. Spatial Variation of Temperature and Precipitation

**Figure 8:**
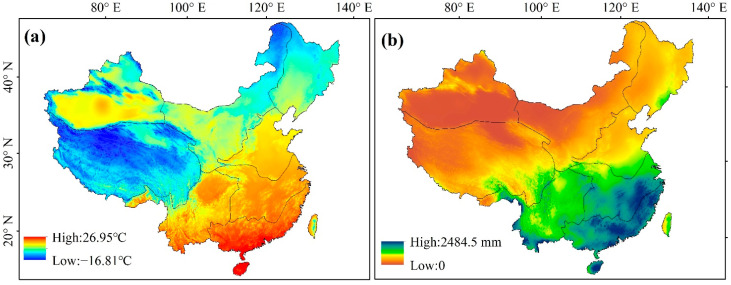
Spatial distribution of average annual temperature and precipitation from 1982 to 2018: (**a**) temperature; (**b**) precipitation.

Figure 8 shows the spatial distribution of average temperature and precipitation from 1982 to 2018, revealing patterns of strong spatial heterogeneity. Over the study period, China has warmed at a rate of 0.38 °C·10 a^−1^, and the temperature has gradually increased from the northwest to the southeast. Among them, the average temperatures of NN, YG, CJ, and SE were significantly higher than those of XJ, QZ, NW, and NE. SE had the highest average annual temperature (19.43 °C), and QZ the lowest (−1.93 °C), with a local peak in temperature in the Tarim Basin of southern XJ. The annual precipitation in southeast China was significantly higher than in the northwest, whereas the annual precipitation decreased from the southeast to the northwest in the study area. SE (1557.35 mm) and CJ (1320.43 mm) witnessed the maximum average annual precipitation levels, while NN (665.38 mm) and NE (575.27 mm) displayed relatively lower levels, and the averages of XJ (151.69 mm), QZ (414.90 mm), and NW (332.53 mm) were the lowest.

**Figure 9:**
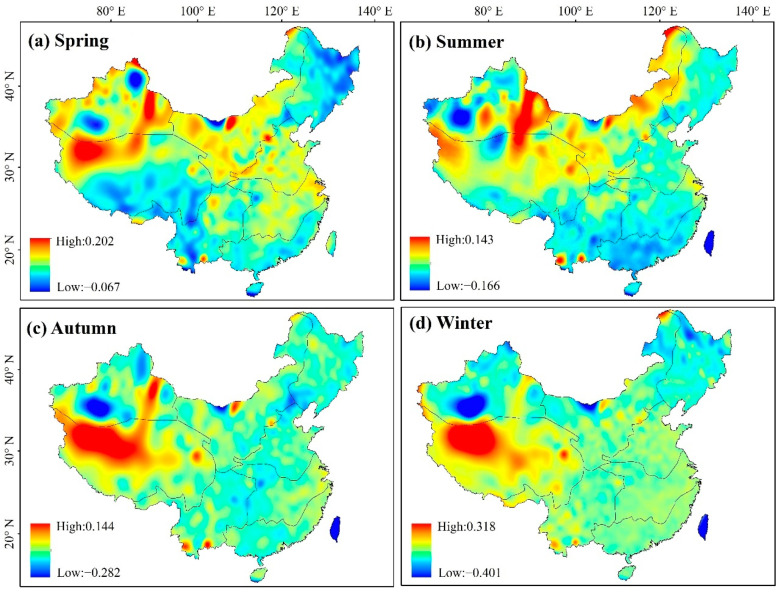
Spatial distribution of seasonal variation trend of temperature from 1982 to 2018.

Figure 9 shows the seasonal spatial distribution of temperature trends from 1982 to 2018. Overall temperatures of the study area increased, with temperatures of spring and summer showing a significant upward trend (rates of 0.05 and 0.04 °C·a^−1^, respectively). The change rate in summer ranged from −0.17 to 0.1 °C·a^−1^, with increasing trends being most apparent in east XJ, NW, and QZ. The autumn and winter rates of change spanned from −0.28 to 0.14 °C·a^−1^ and −0.40 to 0.32 °C·a^−1^, respectively, with a relatively similar spatial distribution between them. The temperature of the Taklamakan Desert in XJ decreased slightly, and western QZ showed a warming trend. Seasonally, the temperatures in different regions increased, albeit to variable degrees.

**Figure 10:**
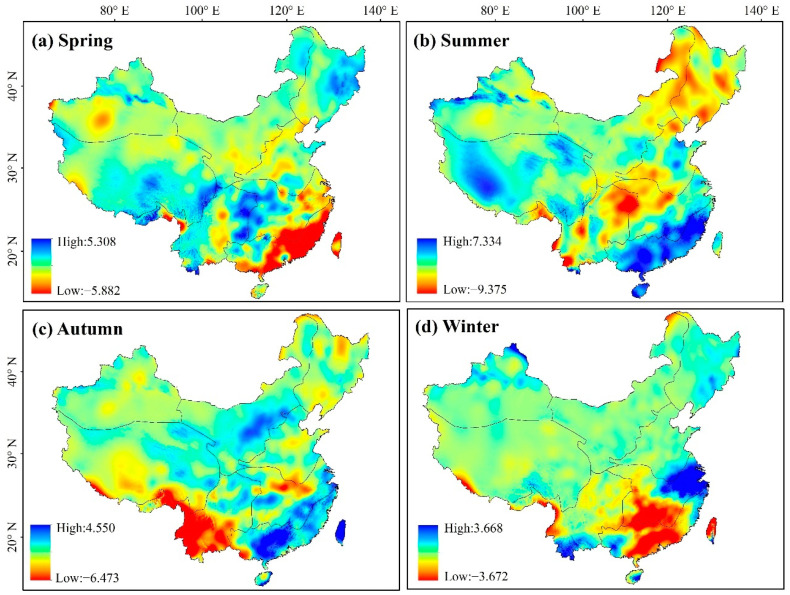
Spatial distribution of seasonal variation trend of precipitation from 1982 to 2018.

The spatial distribution of precipitation trends across the seasons was also variable (Figure 10), with SE precipitation levels displaying the greatest levels of seasonal heterogeneity. The rate of change in SE during spring ranged from −5.88 to 5.31 mm·a^−1^, showing an overall decreasing trend. The highest average summer variation rate was 0.31 mm·a^−1^, with significantly increased precipitation rates recorded in portions of XJ (0.496 mm·a^−1^), QZ (1.223 mm·a^−1^), SE (3.150 mm·a^−1^), and south CJ (0.307 mm·a^−1^). The autumn rate of change ranged from −6.48 to 4.55 mm·a^−1^, with an apparent decreasing trend in southeastern YG and a significant increase in NW. The average change rate was 0.16 mm·a^−1^ in winter, with the precipitation in XJ, NW, and NE showing an increasing trend while that of YG, SE, and south CJ decreased. Therefore, northern China was less rainy, southern China was rainier, and precipitation in the western part of the study area increased significantly over the entire study period.

### 3.4. Correlation between NDVI Dynamics and Climatic Variations

**Figure 11:**
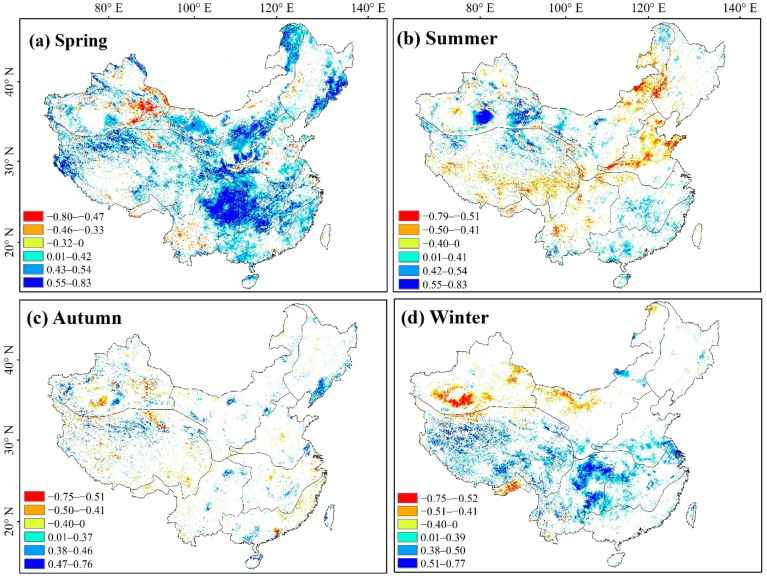
Seasonal correlation analyses (α = 0.05) between vegetation and temperature.

**Figure 12:**
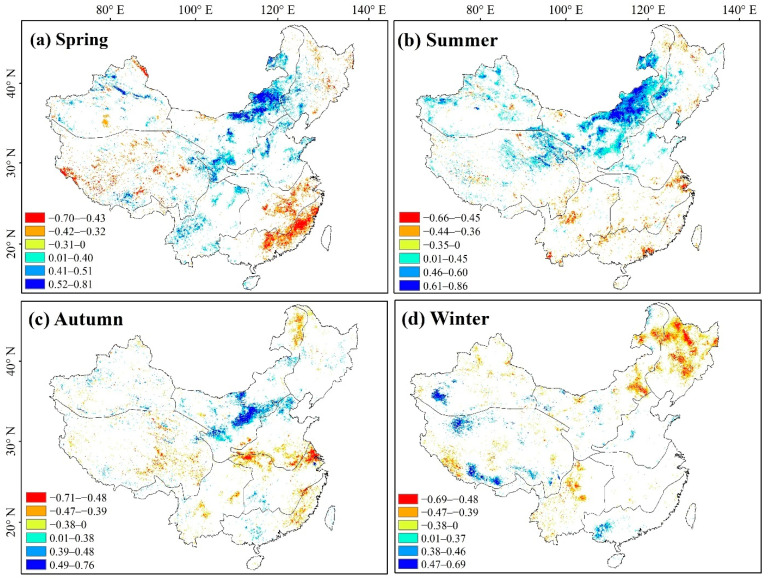
Seasonal correlation analyses (α = 0.05) between vegetation and precipitation.

By calculating the correlation coefficient between NDVI, precipitation, and temperature, the relationship between vegetation and these climatic factors is shown in Figure 11 and Figure 12 and Table 4. Seasonally, a significant positive correlation (R > 0; *p* < 0.05) was shown in spring between NDVI and temperature, which account for 35.75% of total pixels, implying that the promotion effect of temperature on vegetation growth in spring was significantly higher than that of the other three seasons. The percentage of the study area with a significant negative correlation between NDVI and temperature in summer (11.20%) was higher than that with a significant positive correlation (10.62%). The area with a significant negative correlation (R < 0; *p* < 0.05) was mainly distributed between NN (R_NDVI-tem_ = −0.379), northern QZ (R_NDVI-tem_ = −0.051), YG (R_NDVI-tem_ = −0.162), and some areas of NW (R_NDVI-tem_ = −0.078), where increasing temperatures and evapotranspiration levels seemed to have indirectly inhibited the growth of vegetation. The significant correlation between NDVI and temperature in autumn was weak, while the pixels with a significant positive correlation between NDVI and temperature in winter (R > 0; *p* < 0.05) accounted for 14.29%, particularly in QZ (R_NDVI-tem_ = 0.339), YG (R_NDVI-tem_ = 0.328), and CJ (R_NDVI-tem_ = 0.402).

From the correlation analysis results of NDVI and precipitation (Figure 12), it can be concluded that the pixels with a significant positive correlation (R > 0; *p* < 0.05) between NDVI and precipitation in spring, which accounted for 10.13% across China, were mainly concentrated in NW (R_NDVI-pre_ = 0.429) and NN (R_NDVI-pre_ = 0.394). The pixels with a significant negative correlation (R < 0; *p* < 0.05) accounted for 5.32% across China and were mainly distributed in CJ, SE, and some areas of QZ. The count of positive correlation between NDVI and precipitation (R > 0; *p* < 0.05) pixels in summer (13.51%) was significantly higher than that of significant negative correlation (3.71%), with the former mainly distributed in NW (R_NDVI-pre_ = 0.460), XJ (R_NDVI-pre_ = 0.347), NN (R_NDVI-pre_ = 0.343), and QZ (R_NDVI-pre_ = 0.258). The western part of the study area experienced high temperatures and little rainfall year-round, and the observed increase in summer precipitation promoted vegetation growth in these areas with less precipitation. In winter, NDVI significantly negatively correlated with precipitation (R < 0; *p* < 0.05), accounting for 8.59% across China and mainly distributed in NE (R_NDVI-pre_ = −0.410), NW (R_NDVI-pre_ = −0.256), XJ (R_NDVI-pre_ = −0.027), CJ (R_NDVI-pre_ = −0.241), and YG (R_NDVI-pre_ = −0.365), thus indicating that the increase in precipitation in winter inhibited vegetation growth in some areas.

Correlation analyses were performed on NDVI, temperature, and precipitation across each season (Table 4). A notably positive correlation between NDVI and temperature was revealed (R = 0.815, *p* < 0.01), although that between NDVI and precipitation was comparatively weak (R = 0.299). A significant correlation between NDVI and temperature was shown in spring (*p* < 0.01), while that with precipitation was weak; therefore, it was determined that the recovery of temperature in spring was beneficial to vegetation growth. In summer and autumn, NDVI positively correlated with temperature and inversely correlated with precipitation. Summer precipitation levels were higher, although they fluctuated greatly, indicating that the increase in precipitation inhibited vegetation growth. In winter, NDVI significantly positively correlated with average temperatures (*p* < 0.01) and precipitation (*p* < 0.05), while it negatively correlated with average winter precipitation. Winter temperatures were lower, and precipitation appeared to indirectly inhibit the growth of vegetation. To sum up, the vegetation growth in China responded more strongly to temperature than precipitation.

### 3.5. Residual Trend Analysis

**Figure 13:**
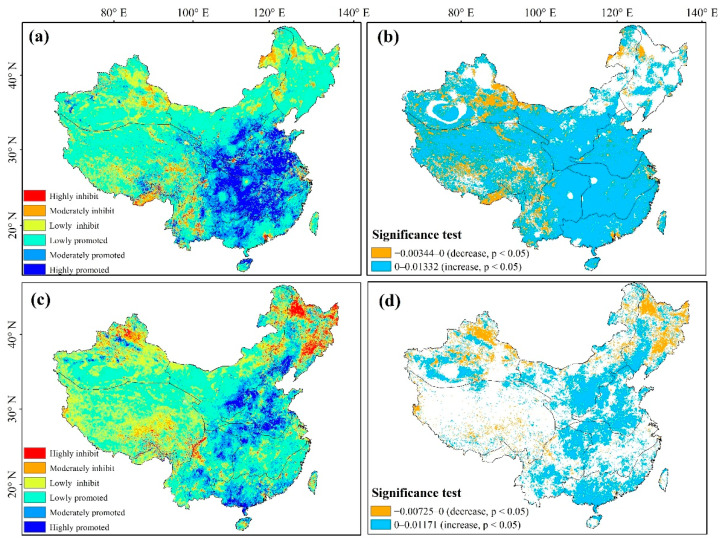
Sen trend analysis and M–K significance test of NDVI under the impacts of climate change (**a**,**b**) and human activities (**c**,**d**).

Figure 13 shows the trend patterns and significance of NDVI_res_ under the impacts of climate change and human activities from 1982 to 2018. Considering the impact of climate factors, 67.72% of the areas showed an increasing trend in NDVI, of which 56.20% showed a significant upward trend (*p* < 0.05). The areas with significant increases in NDVI_pre_ values were mainly concentrated in NW, NN, YG, CJ, and SE. NDVI_pre_ decreased in 32.28% of the areas, with this decrease being significant in 16.4% (*p* < 0.05), particularly in the east of XJ. Over the study area, 49.89% of NDVIres showed an increasing trend, of which a significant increase (*p* < 0.05) was recorded across 22.91% of the total area (mean increase rate, 0.17%·10 a^−1^). The areas with significant increases in NDVI_res_ affected by human activities were mainly concentrated in the southwest of NE, NW, NN, YG, northwest CJ, SE, and south XJ. Alternatively, NDVI_res_ decreased in 50.11% of the regions, with this decrease being significant (*p* < 0.05) in 9.97% of this area, particularly in northern XJ, southern QZ, Daxing’anling, and NE.

**Figure 14:**
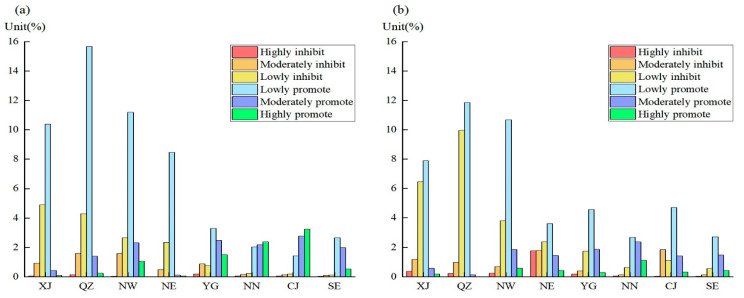
Classified statistics of impacts of climate change (**a**) and human activities (**b**) in eight subregions.

According to the impact degree of climate change and human activities, eight subareas were classified and counted (Figure 14). Climate change has highly promoted the growth of vegetation in CJ (3.24%), YG (1.50%), and NW (1.07%) and lowly promoted the growth of vegetation in QZ (15.67%) and NW (11.20%). Human activities have highly promoted the growth of vegetation in NN (1.11%) and lowly promoted the growth of vegetation in QZ (11.85%) and NW (10.68%) but highly inhibited the growth of vegetation in NE (1.78%), XJ (0.37%), NW (0.23%), and QZ (0.21%). Therefore, emergency mitigation actions are required in areas (NE, XJ, NW, and QZ) where vegetation growth is highly inhibited.

## 4. Discussion

### 4.1. Vegetation and Climate Change Dynamics, 1982–2018

Novel long-term NDVI time series were generated by reconstructing datasets of GIMMS and MODIS following S-G filtering to eliminate noise and reduce error in the remote sensing data in this study. Results revealed that the vegetation growth rate in China has shown a trend of gradual improvement (0.5%·10 a^−1^) over the past 37 years (1982–2018), notably consistent with NDVI trends obtained in other global arid regions and Central Asia [24,37]. Therefore, it was concluded that the intensity of photosynthesis is increasing under the backdrop of climate change, as the vegetation displayed a marked greening trend. The significantly improved areas of vegetation cover in China were greater than those of the significantly degraded coverage from 1982 to 2018, with Central and Eastern China showing the most remarkable significant trends of improvement. Research has shown that global vegetation greenness has been increasing under long-term vegetation monitoring [37,38]. Data from various NASA Earth satellites have shown that certain anthropogenic activities, such as afforestation and agriculture in China and India, dominate the global greening process, supporting the gradual improvement of vegetation coverage observed here across China [38]. Moreover, temperature trends have played a key role in ecological protection, biodiversity, and climate change [38,39]. Over the study period, China has been warming at a rate of 0.38 °C·10 a^−1^, confirming that the patterns observed over the study area are consistent with the general global trends. Notably, 37 years of decreased rain in the north, increased rain in the south, and significantly increased in precipitation to the west of China were also observed. Based on the study of Li et al., this paper extended the GIMMS time series, analyzed the dynamic changes of vegetation and its correlation with climate from different regional units and finer time scales (seasons), and clarified the characteristics of seasonal changes of vegetation in China and its seasonal response to climate. This study found that the vegetation in QZ and the Greater Hinggan Mountains in the northeastern part of Inner Mongolia shows a significant downward trend, and the results were consistent with the trend observed by Li et al. The eastern part of Inner Mongolia shows a significant downward trend, and the results were consistent with the trend observed by Li et al. [27].

### 4.2. Influences of Climate Change and Human Activities on Vegetation Growth

Vegetation growth is jointly influenced by climate change and human activity [40,41]. A significant positive correlation (R > 0; *p* < 0.05) was found between 35.75% of vegetated areas and temperature in spring, with these being significantly higher than those observed in the other three seasons, likely due to the optimization of spring atmospheric and ground temperatures, as well as the transpiration levels for plant growth [42,43]. Summers were hot and rainy, and although the increase in temperature significantly inhibited vegetation growth (R > 0; *p* < 0.05) in NN, southern QZ, YG, and parts of NW, the increase in precipitation significantly promoted vegetation growth (R > 0; *p* < 0.05) in areas with less precipitation, such as NW, XJ, NN, and QZ. Due to the rising summer temperatures, photosynthesis is weakened, respiration is enhanced, the plant water balance is disrupted, transpiration is promoted, and more infrared rays are reflected in summer than in winter. When temperatures exceeded the upper limits of suitable temperature zones for vegetation, injurious effects were imparted on plants, hindering their growth and development, especially during the flowering and fruiting period, when they are most vulnerable. Thus, the higher are the temperatures, the greater is the injurious effect on the plants. An increase in summer precipitation can promote vegetation growth in areas with high temperatures and limited rainfall year-round, as optimal water and heat conditions benefit vegetative growth. When coupled with increased efforts to protect and restore ecosystems, such as afforestation, these factors have strongly contributed to promoting the good ecological quality of vegetation [14,44]. Conversely, the vegetation in NE, northern XJ, and the NW Xiaoxing’anling areas has significantly degraded in winter. Under the influence of global climate change, winter precipitation significantly suppressed vegetation growth (R > 0; *p* < 0.05) in NE (R_NDVI-pre_ = −0.410), XJ (R_NDVI-pre_ = −0.027), and NW (R_NDVI-pre_ = −0.256). In winter, lower temperatures and increased precipitation seemed to have inhibited vegetation growth in western China.

Climate change and human activities exert two-way moderating effects on vegetation [44]. A previous study showed that human activities and overexploitation can degrade vegetation in Xinjiang [45]. The present study shows that 22.91% of the total area witnessed a significant increase in vegetation coverage (*p* < 0.05) under the effects of human activities from 1982 to 2018, whereas 9.97% of the area experienced significantly decreased vegetation cover (*p* < 0.05), thereby indicating a positive effect of human activities on vegetation changes. Ecological projects, such as afforestation, construction of the Three-North Shelterbelt, wildlife protection policies, conversion of farmland to forests, and nature reserve development projects, thus appear to have yielded a net positive impact on vegetation growth [46,47,48]. Notably, the vegetation coverage in northern XJ, NE, and NW semiarid regions showed a downward trend, likely a result of climate change, in addition to the destruction of vegetation by human activities, such as overgrazing and cultivation, leading to such effects as the desertification of grasslands, degradation of vegetation, decline of productivity, enhanced wind erosion of land, and aggravation of soil erosion [49,50]. Human activities highly inhibited the growth of vegetation in NE (1.78%), XJ (0.37%), NW (0.23%), and QZ (0.21%). Overexploitation of resources and rapid urban development under the disturbance of human activities have also contributed to the severe degradation of vegetation [14,41]. Therefore, emergency mitigation actions are required in NE, XJ, NW, and QZ.

### 4.3. Study Limitations

Limited by the time range of data resources, the present research employed multisource remote sensing data to merge and extend time series interpolation, providing reliable data support for ecosystem research. The vegetation changes observed here were influenced by a combination of natural and anthropogenic factors. However, the present study only considered the effects of temperature, precipitation, and human activities on vegetation change. Therefore, subsequent analyses shall compile various climatic factors affecting vegetation growth for screening via a stepwise regression method, thus revealing the primary factors underlying vegetation growth. Considering the complexity of model coupling, a black box structural equation model (i.e., neural network) will be used to analyze the joint influence of anthropogenic activity and natural elements on vegetation change. The socioeconomic and natural element data of the key ecological restoration project areas will be collected and collated. Structural equation models will be constructed separately to conduct comparative studies to clarify the degrees of influence in each region, providing a certain reference basis for constructing ecological projects [51,52,53,54].

## 5. Conclusions

The spatiotemporal characteristics of NDVI and climate factors in China were assessed here using GIMMS NDVI3g data from 1982 to 2018. Results showed that:

1. Vegetation and climate change throughout China displayed marked spatiotemporal heterogeneity. From 1982 to 2018, the vegetation showed a gradually increasing trend at a rate of 0.5%·10 a^−1^. Therefore, it was concluded that the vegetation area in the study area that is significantly improving (37.15%, *p* < 0.05) was statistically more extended than that undergoing significant degradation (7.46%, *p* < 0.05).
2. From 1982 to 2018, the study area showed a warming trend at a rate of 0.38 °C·10 a^−1^, whereas precipitation showed a stronger spatial heterogeneity, gradually decreasing from the southeast to the northwest, with notably less rain in the north compared to the south.
3. Temperature significantly affected the vegetation growth in spring (R > 0; *p* < 0.05); however, the increase in summer temperatures significantly inhibited the growth (R < 0; *p* < 0.05) in NN (R_NDVI-tem_ = −0.379), QZ (R_NDVI-tem_ = −0.051), YG (R_NDVI-tem_ = −0.162), some parts of NW (R_NDVI-tem_ = −0.078), and other areas. The regions with significantly positive correlations between spring NDVI, summer NDVI, and precipitation (R > 0; *p* < 0.05) accounted for 10.13% and 13.51% of the study area, respectively. In winter, NDVI significantly negatively correlated with precipitation (R > 0; *p* < 0.05) and was mostly distributed in NE (R_NDVI-pre_ = −0.410), NW (R_NDVI-pre_ = −0.256), and YG (R_NDVI-pre_ = −0.365). Therefore, it was also concluded that the increase in winter precipitation inhibited vegetation growth in some areas.
4. Residual trend analysis showed that human activities had a double-edged effect on vegetation change, where growth across 22.91% of the study area was significantly promoted by anthropogenic activity (*p* < 0.05) and inhibited across 9.97% of the study area (*p* < 0.05).

## Figures and Tables

**Table 1:** Precision validation of GIMMS NDVI3g and MOD13A2 NDVI data before and after S–G filter reconstruction, 2001–2015.

	GIMMS	GIMMS_S-G_	MOD13A2	MOD13A2_S-G_
GIMMS	1	0.994 **	0.941 **	0.962 **
GIMMS_S-G_		1	0.929 **	0.966 **
MOD13A2			1	0.961 **
MOD13A2_S-G_				1

Note: **, *p* < 0.01.

**Table 2:** Area NDVI statistics for each grade.

Level	NDVI < 0.1	0.1 < NDVI < 0.2	0.2 < NDVI < 0.3	0.3 < NDVI < 0.4	0.4 < NDVI < 0.5	0.5 < NDVI < 0.6	NDVI > 0.6
Area	21.42%	15.64%	10.22%	10.13%	12.43%	13.67%	16.5%

**Table 3:** NDVI trends in China.

S_NDVI_	Z	NDVI Trend	Annual	Spring	Summer	Autumn	Winter
≥0.0005	≥1.96	Significantly improved	37.15	34.59	21.85	22.73	25.02
≥0.0005	−1.96–1.96	Slightly improved	3.00	12.93	12.12	13.42	8.16
−0.0005–0.0005	−1.96–1.96	Stable	50.52	41.63	43.43	44.34	42.88
<−0.0005	−1.96–1.96	Slightly degraded	1.88	4.86	10.96	10.57	6.52
<−0.0005	≤−1.96	Severely degraded	7.46	5.98	11.64	8.94	17.42

Note: Stable means S_NDVI_ (−0.0005, 0.0005) and Z (−1.91, 1.96).

**Table 4:** CC of NDVI with temperature and precipitation.

	Spring NDVI	Summer NDVI	Autumn NDVI	Winter NDVI	Annual NDVI
Spring average temperature	0.707 **	−0.068	0.116	0.438 **	0.733 **
Summer average temperature	0.655 **	0.033	0.155	0.471 **	0.720 **
Autumn average temperature	0.522 **	0.233	0.279	0.234	0.593 **
Winter average temperature	0.529 **	0.007	0.021	0.264	0.540 **
Annual average temperature	0.766 **	0.054	0.164	0.437 **	0.815 **
Spring average precipitation	0.273	−0.033	−0.003	0.246	0.266
Summer average precipitation	0.312	−0.195	−0.017	0.354 *	0.287
Autumn average precipitation	−0.137	−0.323	−0.176	0.254	0.004
Winter average precipitation	0.159	0.025	0.113	−0.043	0.129
Annual average precipitation	0.261	−0.258	−0.060	0.395 *	0.299

Note: *, *p* < 0.05; **, *p* < 0.01.

## References

[1] IPCC (2021). Climate Change 2021: The Physical Science Basis. Contribution of Working Group I to the Sixth Assessment Report of the Intergovernmental Panel on Climate Change.

[2] Buitenwerf R., Rose L., Higgins S.I. (2015). Three decades of multi-dimensional change in global leaf phenology. Nat. Clim. Chang..

[3] Hou W.J., Gao J.B., Wu S.H., Dai E.F. (2015). Interannual variations in growing-season NDVI and its correlation with climate variables in the southwestern karst region of China. Remote Sens..

[4] Li C., Qi J., Yang L., Wang S., Yang W., Zhu G., Zou S., Zhang F.J. (2014). Regional vegetation dynamics and its response to climate change—A case study in the Tao River Basin in Northwestern China. Environ. Res. Lett..

[5] Xu S., Yu Z., Lettenmaier D.P., McVicar T.R., Ji X.J. (2020). Elevation-dependent response of vegetation dynamics to climate change in a cold mountainous region. Environ. Res. Lett..

[6] Grodek T., Morin E., Helman D., Lensky I., Dahan O., Seely M., Enzel Y. (2020). Eco-hydrology and geomorphology of the largest floods along the hyperarid Kuiseb River, Namibia. J. Hydrol.

[7] Yu L.X., Liu T.X., Bu K., Yan F.Q., Yang J.C., Chang L.P., Zhang S.W. (2017). Monitoring the long term vegetation phenology change in Northeast China from 1982 to 2015. Sci. Rep..

[8] Nemani R.R., Keeling C.D., Hashimoto H., Jolly W.M., Piper S.C., Tucker C.J., Myneni R.B., Running S.W. (2003). Climate-driven increases in global terrestrial net primary production from 1982 to 1999. Science.

[9] Zheng K., Wei J.Z., Pei J.Y., Cheng H., Zhang X.L., Huang F.Q., Li F.M., Ye J.S. (2019). Impacts of climate change and human activities on grassland vegetation variation in the Chinese Loess Plateau. Sci. Total Environ..

[10] Zhu X., Hu Y., Gao B.J. (2011). Influence of Environment of Forest-Steppe Ecotone on Soil Arthropods Community in Northern Hebei, China. Proced. Environ. Sci..

[11] Chapungu L., Nhamo L., Gatti R.C. (2020). Estimating biomass of savanna grasslands as a proxy of carbon stock using multispectral remote sensing. Remote Sens. Appl. Soc. Environ..

[12] Campagnolo M.L., Libonati R., Rodrigues J.A., Pereira J.J. (2021). A comprehensive characterization of MODIS daily burned area mapping accuracy across fire sizes in tropical savannas. Remote Sens. Environ..

[13] Moreno R., Ojeda N., Azocar J., Venegas C., Inostroza L.J.U.F., Greening U. (2020). Application of NDVI for identify potentiality of the urban forest for the design of a green corridors system in intermediary cities of Latin America: Case study, Temuco, Chile. Urban For. Urban Green..

[14] Chu H.S., Venevsky S., Wu C., Wang M.H. (2019). NDVI-based vegetation dynamics and its response to climate changes at Amur-Heilongjiang River Basin from 1982 to 2015. Sci. Total Environ..

[15] Gao S.Q., Dong G.T., Jiang X.H., Nie T., Yin H.J., Guo X.W. (2021). Quantification of Natural and Anthropogenic Driving Forces of Vegetation Changes in the Three-River Headwater Region during 1982–2015 Based on Geographical Detector Model. Remote Sens..

[16] Kang Y., Guo E.L., Wang Y.F., Bao Y.L., Bao Y.H., Mandula N. (2021). Monitoring Vegetation Change and Its Potential Drivers in Inner Mongolia from 2000 to 2019. Remote Sens..

[17] Chen T., Bao A.M., Jiapaer G., Guo H., Zheng G.X., Jiang L.L., Chang C., Tuerhanjiang L. (2019). Disentangling the relative impacts of climate change and human activities on arid and semiarid grasslands in Central Asia during 1982–2015. Sci. Total Environ..

[18] Jin Z., Liang W., Yang Y., Zhang W., Yan J., Chen X., Li S., Mo X.J. (2017). Separating vegetation greening and climate change controls on evapotranspiration trend over the Loess Plateau. Sci. Rep..

[19] Feng Z., Zhang P. (2004). “Grain for Green” Policy and its impacts on grain supply in west China. China’s West Reg. Develop..

[20] Sun W.Y., Song X.Y., Mu X.M., Gao P., Wang F., Zhao G.J. (2015). Spatiotemporal vegetation cover variations associated with climate change and ecological restoration in the Loess Plateau. Agric. Forest Meteorol..

[21] Markogianni V., Dimitriou E. (2016). Landuse and NDVI change analysis of Sperchios river basin (Greece) with different spatial resolution sensor data by Landsat/MSS/TM and OLI. Desalin. Water Treat..

[22] Hao P.Y., Di L.P., Zhang C., Guo L.Y. (2020). Transfer learning for crop classification with cropland data layer data (CDL) as training samples. Sci. Total Environ..

[23] Maselli F. (2004). Monitoring forest conditions in a protected Mediterranean coastal area by the analysis of multiyear NDVI data. Remote Sens. Environ..

[24] Munyati C., Mboweni G. (2013). Variation in NDVI values with change in spatial resolution for semi-arid savanna vegetation: A case study in northwestern South Africa. Int. J. Remote Sens..

[25] Tarnavsky E., Garrigues S., Brown M.E. (2008). Multiscale geostatistical analysis of AVHRR, SPOT-VGT, and MODIS global NDVI products. Remote Sens. Environ..

[26] Beck H.E., McVicar T.R., van Dijk A.I., Schellekens J., de Jeu R.A., Bruijnzeel L.A. (2011). Global evaluation of four AVHRR–NDVI data sets: Intercomparison and assessment against Landsat imagery. Remote Sens. Environ..

[27] Li Y., Xie Z.X., Qin Y.C., Zheng Z.C. (2019). Estimating relations of vegetation, climate change, and human activity: A case study in the 400 mm annual precipitation fluctuation zone, China. Remote Sens..

[28] Zhang Y., Song C., Band L.E. (2017). Reanalysis of global terrestrial vegetation trends from MODIS products: Browning or greening?. Remote Sens. Environ..

[29] Fensholt R., Proud S.R. (2012). Evaluation of earth observation based global long term vegetation trends—Comparing GIMMS and MODIS global NDVI time series. Remote Sens. Environ..

[30] Savitzky A. (1964). Smoothing and differentiation of data by simplified least squares procedures. Anal. Chem..

[31] Mao D., Wang Z., Luo L., Ren C. (2012). Integrating AVHRR and MODIS data to monitor NDVI changes and their relationships with climatic parameters in Northeast China. Intern. J. Appl. Earth Obs. Geoinform..

[32] Du J.Q., Shu J.M., Wang Y.H., Li Y.C., Guo Y. (2014). Comparison of GIMMS and MODIS normalized vegetation index composite data for Qing-Hai-Tibet Plateau. Ying Yong Sheng Tai Xue Bao.

[33] Tucker C., Pinzon J., Brown M., Slayback D., Pak E., Mahoney R., Vermote E., El Saleous N. (2005). An extended AVHRR 8-km NDVI dataset compatible with MODIS and SPOT vegetation NDVI data. Intern. J. Remote Sens.

[34] Zhang L.S., Luo H.J., Zhang X.Z. (2022). Land-Greening Hotspot Changes in the Yangtze River Economic Belt during the Last Four Decades and Their Connections to Human Activities. Land.

[35] Evans J., Geerken R. (2004). Discrimination between climate and human-induced dryland degradation. J. Arid. Environ..

[36] Wen Z.F., Wu S.J., Chen J.L., Lu M.Q. (2017). NDVI indicated long-term interannual changes in vegetation activities and their responses to climatic and anthropogenic factors in the Three Gorges Reservoir Region, China. Sci. Total Environ..

[37] Guo J.T., Hu Y.M., Xiong Z.P., Yan X.L., Ren B.H., Bu R.C. (2017). Spatiotemporal variations of growing-season NDVI associated with climate change in Northeastern China’s permafrost zone. Pol. J. Environ. Stud..

[38] NASA Human Activity in China and India Dominates the Greening of Earth, NASA Study Shows. https://www.nasa.gov/feature/ames/human-activity-in-china-and-india-dominates-the-greening-of-earth-nasa-study-shows.

[39] Chen C., Park T., Wang X.H., Piao S.L., Xu B.D., Chaturvedi R.K., Fuchs R., Brovkin V., Ciais P., Fensholt R. (2019). China and India lead in greening of the world through land-use management. Nat. Sustain..

[40] Tao Y., Wu G.L., Zhang Y.M. (2017). Dune-scale distribution pattern of herbaceous plants and their relationship with environmental factors in a saline–alkali desert in Central Asia. Sci. Total Environ..

[41] Li Y., Zheng Z.C., Qin Y.C., Rong P.J. (2021). Relative contributions of natural and man-made factors to vegetation cover change of environmentally sensitive and vulnerable areas of China. J. Clean. Prod..

[42] Wang X., Piao S., Ciais P., Li J., Friedlingstein P., Koven C., Dickinson C.J. (2011). Spring temperature change and its implication in the change of vegetation growth in North America from 1982 to 2006. Proc. Natl. Acad. Sci. USA.

[43] Li Y., Qin Y.C., Rong P.J. (2022). Evolution of potential evapotranspiration and its sensitivity to climate change based on the Thornthwaite, Hargreaves, and Penman-Monteith equation in environmental sensitive areas of China. Atmos. Res..

[44] Jiang L.L., Jiapaer G., Bao A.M., Guo H., Ndayisaba F. (2017). Vegetation dynamics and responses to climate change and human activities in Central Asia. Sci. Total Environ..

[45] Liu Y., Li L.H., Chen X., Zhang R., Yang J.M. (2018). Temporal-spatial variations and influencing factors of vegetation cover in Xinjiang from 1982 to 2013 based on GIMMS-NDVI3g. Glob. Planet. Chang..

[46] Li Y., Xie Z.X., Qin Y.C., Sun Y.Y. (2019). Temporal-spatial variation characteristics of soil erosion in the Pisha Sandstone Area, Loess Plateau, China. Pol. J. Environ. Stud..

[47] Liu H., Li X.J., Mao F.J., Zhang M., Zhu D., He S.B., Huang Z.H., Du H.Q. (2021). Spatiotemporal evolution of fractional vegetation cover and its response to climate change based on MODIS data in the subtropical region of China. Remote Sens..

[48] Enping Y., Hui L., Yongfeng D., Chaozong X.J.A. (2014). The spatiotemporal changes of vegetation cover in Beijing-Tianjin sandstorm source control region during 2000–2012. Shengtai Xuebao Acta Ecol. Sin..

[49] Wang Z., Shi Q.S., Wang T., Shi Q.D., Chang S.L., Zhang L.B. (2011). Spatial-temporal characteristics of vegetation cover change in mountain-oasis-desert system of Xinjiang from 1982 to 2006. J. Nat. Resour..

[50] Zhao Y., Yu Z.C., Chen F.H. (2009). Spatial and temporal patterns of Holocene vegetation and climate changes in arid and semi-arid China. Quatern. Int..

[51] Li H., Zhang H.Y., Li Q.X., Zhao J.J., Guo X.Y., Ying H., Deng G.R., Wu R.H., Wang S.L. (2021). Vegetation productivity dynamics in response to climate change and human activities under different topography and land cover in Northeast China. Remote Sens..

[52] Zhang K., Kimball J.S., Nemani R.R., Running S.W., Hong Y., Gourley J.J., Yu Z.B. (2015). Vegetation greening and climate change promote multidecadal rises of global land evapotranspiration. Sci. Rep..

[53] Meng M., Huang N., Wu M.Q., Pei J., Wang J., Niu Z. (2019). Vegetation change in response to climate factors and human activities on the Mongolian Plateau. Peerj.

[54] Xu H.J., Wang X.P., Yang T.B. (2017). Trend shifts in satellite-derived vegetation growth in Central Eurasia, 1982–2013. Sci. Total Environ..

